# Morphology-guided transcriptomic analysis of human pancreatic cancer organoids reveals microenvironmental signals that enhance invasion

**DOI:** 10.1172/JCI162054

**Published:** 2023-04-17

**Authors:** Yea Ji Jeong, Hildur Knutsdottir, Fatemeh Shojaeian, Michael G. Lerner, Maria F. Wissler, Elodie Henriet, Tammy Ng, Shalini Datta, Bernat Navarro-Serer, Peter Chianchiano, Benedict Kinny-Köster, Jacquelyn W. Zimmerman, Genevieve Stein-O’Brien, Matthias M. Gaida, James R. Eshleman, Ming-Tseh Lin, Elana J. Fertig, Andrew J. Ewald, Joel S. Bader, Laura D. Wood

**Affiliations:** 1 Department of Pathology, Sol Goldman Pancreatic Cancer Research Center, Johns Hopkins University School of Medicine, Baltimore, Maryland, USA.; 2 Department of Biomedical Engineering, Johns Hopkins University Whiting School of Engineering, Baltimore, Maryland, USA.; 3 Department of Physics and Astronomy, Earlham College, Richmond, Indiana, USA.; 4 Department of Cell Biology,; 5 Department of Surgery,; 6 Department of Oncology, Sidney Kimmel Comprehensive Cancer Center, and; 7 Convergence Institute, Johns Hopkins University School of Medicine, Baltimore, Maryland, USA.; 8 Department of Pathology, University of Mainz, Mainz, Germany.; 9 TRON, Translational Oncology at the University Medical Center, Mainz, Germany.; 10 Research Center for Immunotherapy, University Medical Center Mainz, Johannes Gutenberg University Mainz, Mainz, Germany.; 11 Department of Applied Mathematics and Statistics, Johns Hopkins University, Baltimore, Maryland, USA.

**Keywords:** Gastroenterology, Oncology, Cell migration/adhesion, Molecular genetics

## Abstract

Pancreatic ductal adenocarcinoma (PDAC) frequently presents with metastasis, but the molecular programs in human PDAC cells that drive invasion are not well understood. Using an experimental pipeline enabling PDAC organoid isolation and collection based on invasive phenotype, we assessed the transcriptomic programs associated with invasion in our organoid model. We identified differentially expressed genes in invasive organoids compared with matched noninvasive organoids from the same patients, and we confirmed that the encoded proteins were enhanced in organoid invasive protrusions. We identified 3 distinct transcriptomic groups in invasive organoids, 2 of which correlated directly with the morphological invasion patterns and were characterized by distinct upregulated pathways. Leveraging publicly available single-cell RNA-sequencing data, we mapped our transcriptomic groups onto human PDAC tissue samples, highlighting differences in the tumor microenvironment between transcriptomic groups and suggesting that non-neoplastic cells in the tumor microenvironment can modulate tumor cell invasion. To further address this possibility, we performed computational ligand-receptor analysis and validated the impact of multiple ligands (TGF-β1, IL-6, CXCL12, MMP9) on invasion and gene expression in an independent cohort of fresh human PDAC organoids. Our results identify molecular programs driving morphologically defined invasion patterns and highlight the tumor microenvironment as a potential modulator of these programs.

## Introduction

Pancreatic ductal adenocarcinoma (PDAC) has a dismal prognosis that is due, at least in part, to its late stage at diagnosis, with almost 90% of patients presenting with metastasis (1, 2). Although the genomic alterations that accumulate during early pancreatic tumorigenesis are well characterized (3–6), the molecular alterations driving metastatic spread are incompletely understood. The lack of metastasis-specific mutations in driver genes when comparing primary to metastatic PDACs suggests that metastasis is not controlled by genetic alterations (7). In recent years, transcriptomic characterization of PDAC has led to the proposal of various molecular subtyping schemes in order to capture the transcriptomic and proteomic heterogeneity of PDAC (8–14). Synthesis of these studies suggests 2 distinct transcriptomic subtypes of PDAC, frequently termed classical and basal, with the basal subtype associated with a poorer outcome (8, 9). Although the basal subtype is enriched in metastatic lesions compared with primary tumors (8), the role of transcriptomic alterations in driving metastasis remains unclear.

Local invasion is the earliest stage in the multistep process of metastatic dissemination (15–17). Traditionally, local invasion was thought to occur via epithelial-mesenchymal transition (EMT), in which carcinoma cells acquire mesenchymal characteristics and disseminate as single cells via activation of a mesenchymal transcriptional program, including Snail, Twist, and Zeb (17, 18). However, an emerging body of evidence suggests an alternate mode of invasion, collective invasion, in which cancer cells retain their epithelial features and disseminate as multicellular clusters (19, 20). While this phenomenon has been challenging to recapitulate in traditional in vitro or in vivo models, recent advances in 3-dimensional organoid models have enabled a real-time observation of collective invasion of carcinomas of the breast and pancreas (21–24). When clusters of freshly harvested PDAC cells are cultured as organoids in collagen I gels, they invade the surrounding matrix, allowing morphological characterization of the invasive phenotype.

Previously, we demonstrated that organoids from surgically resected human PDACs show either a mesenchymal or collective invasion pattern in vitro, characterized by sharp single-cell protrusions or curvilinear multicellular projections, respectively (21). In that study, we identified transforming growth factor β (TGF-β) as a potent inducer of invasion in some organoid cultures, but we also discovered that the molecular drivers of invasion varied between patients. Therefore, comprehensive assessment of transcriptomic alterations in invading organoids from human PDACs is required to elucidate the molecular programs that drive cancer cell invasion.

In this study, we used a collection and sorting pipeline that we developed to isolate organoids based on invasion pattern, enabling us to determine the transcriptomic programs underlying the collective and mesenchymal patterns of invasion. We performed morphology-guided transcriptomic profiling on a cohort of fresh human PDAC organoids, linked our findings to publicly available single-cell RNA-sequencing (scRNA-seq) data from human PDACs, and then validated key results in a second independent cohort of fresh human PDAC organoids. Our results suggest that collective and mesenchymal invasion are orchestrated via 2 distinct transcriptomic programs and influenced by interactions between the tumor cells and cells in the surrounding microenvironment.

## Results

**Figure 1:**
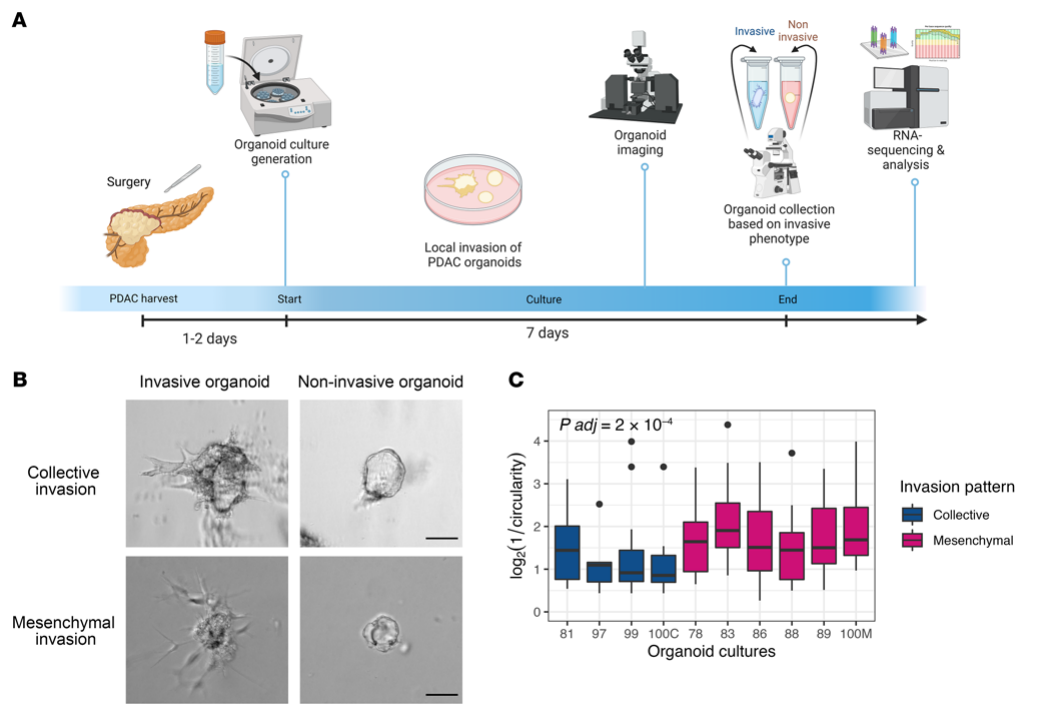
Patient-derived PDAC organoids show 2 morphometrically distinct invasive phenotypes. (**A**) Schematic of PDAC organoid culture generation, collection, and RNA-seq timeline. (**B**) Representative images of collective and mesenchymal invasion. Scale bars: 50 μm. (**C**) Log_2_(1/circularity) of collective and mesenchymal organoids (*n* = 10, adjusted *P* value=2 × 10^–4^ using Wilcoxon’s test). In the box-and-whisker plot, the box outlines represent the 25th to 75th percentiles, horizontal lines represent medians, and whiskers extend to 1.5 × IQR.

To identify the transcriptomic signatures associated with invasion in PDAC, we performed RNA-seq of organoids representing previously described collective and mesenchymal invasion patterns (21), along with paired noninvasive organoids. To do this, we developed a method to isolate organoids based on invasive phenotype for molecular profiling (Figure 1A). Using this method, a total of 11 organoid cultures were generated from surgically resected human PDACs that did not receive neoadjuvant chemotherapy. Among these, 7 cultures showed a predominantly mesenchymal invasion pattern (“mesenchymal organoids”), and 3 showed a predominantly collective invasion pattern (“collective organoids”) (Figure 1B). Intriguingly, 1 tumor gave rise to both collective and mesenchymal organoids, and these invasion patterns were mutually exclusive at the individual organoid level, consistent with our previous work (21). For this organoid culture, the collective and mesenchymal organoids were analyzed separately. Thus, we analyzed a total of 8 mesenchymal organoid cultures and 4 collective organoid cultures with 11 matching noninvasive organoid cultures from the same primary tumors.

The clinical and pathological features of the primary tumors (Supplemental Table 1; supplemental material available online with this article; https://doi.org/10.1172/JCI162054DS1) demonstrate that the pattern of invasion in our organoid model was neither correlated with the size of the primary tumor (*P* = 0.808, Mann-Whitney *U* test) nor with its grade or the presence of lymph node metastasis (*P* = 1.00 and *P* = 0.162, χ^2^ test). To investigate whether driver gene mutations were enriched in PDACs with either invasive phenotype, we performed targeted next-generation DNA sequencing of primary tumor tissue using a panel of 432 cancer-associated genes (Supplemental Table 2). The prevalence of mutations in *KRAS*, *CDKN2A*, *TP53*, and *SMAD4* did not differ between the 2 invasion patterns in our organoid system, and mutations in other genes occurred in 3 or fewer patients (i.e., *ARID1A* and *ARID1B*) (Supplemental Table 3).

To quantitatively compare the 2 distinct invasion patterns and assess the degree of invasiveness, we counted the number of invasive protrusions in each imaged organoid and traced the borders of each organoid to calculate an inverse circularity score for each organoid culture (defined as log_2_[1/circularity]). A high inverse circularity score indicates a deviation from a perfectly round organoid and is a metric of invasiveness. The mesenchymal organoids had a higher mean number of invasive protrusions per organoid compared with the collective organoids (5.3 vs. 2.6, *P* = 7 × 10^–6^). In addition, the mean inverse circularity score of the mesenchymal organoids was significantly higher than that of the collective organoids (1.6 vs. 1.0, Wilcoxon’s *P* = 2 × 10^–4^) (Figure 1C). These results demonstrate morphometric differences between mesenchymal and collective organoids and underscore the qualitatively and quantitatively distinct invasion patterns in our human PDAC organoid model, highlighting this system as a potentially powerful tool for molecular interrogation of PDAC invasion.

**Figure 2:**
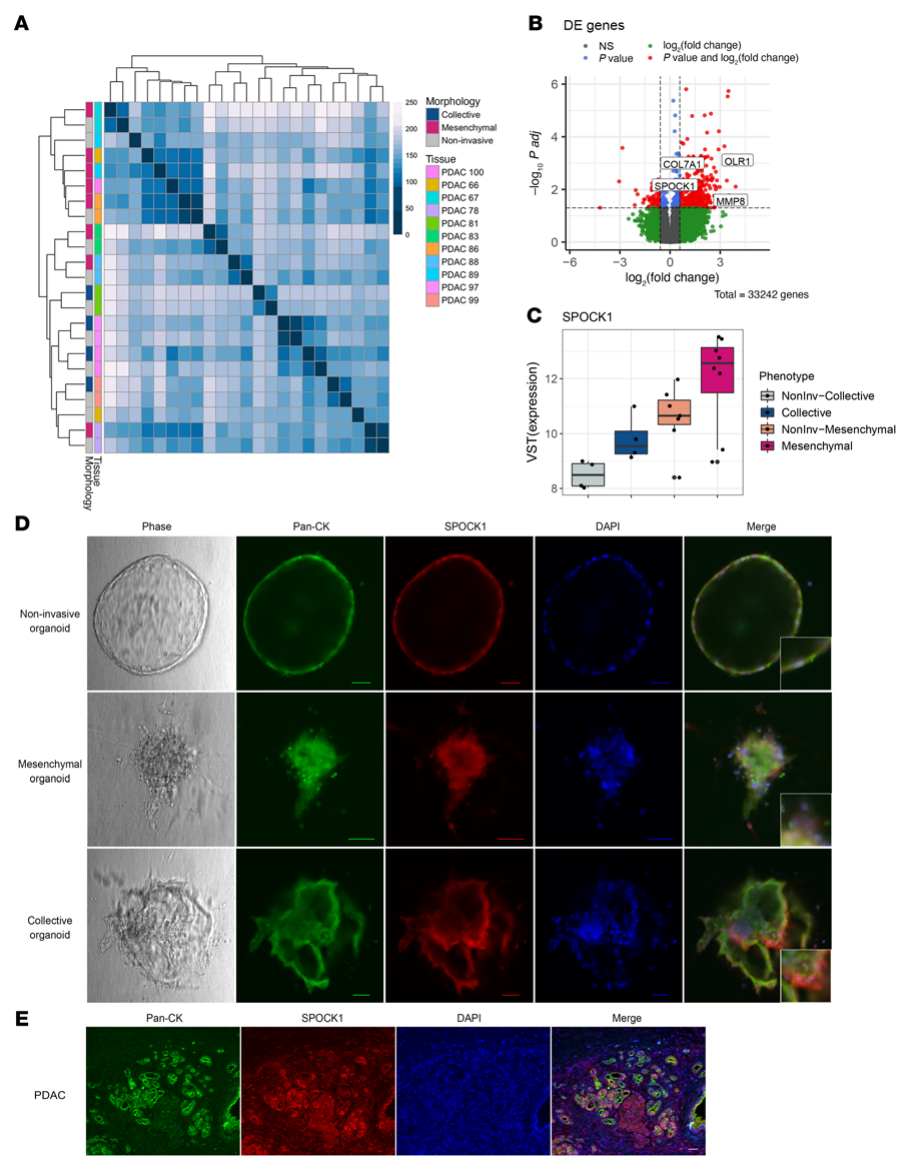
Comparison of transcriptomes of invasive and noninvasive organoids reveals differentially expressed genes that are enhanced in invasive protrusions. (**A**) A sample-to-sample distance plot (Spearman’s correlation) of the variance-stabilizing transform (VST) gene expression for each sample (*n* = 23). (**B**) Differentially expressed (DE) genes between all invasive organoids (*n* = 12) and noninvasive organoids (*n* = 11) (adjusted-*P* threshold of 0.05). *OLR1*, *SPOCK1*, *MMP8*, and *COL7A1* were all upregulated in invasive organoids compared with the noninvasive organoids. (**C**) *SPOCK1* mRNA expression in indicated organoid groups. Each dot represents an organoid culture. In the box-and-whisker plot, the box boundaries represent the 25th to 75th percentiles, horizontal lines represent medians, and whiskers extend to 1.5 × IQR. (**D**) Phase and immunofluorescence images of pan-CK, SPOCK1, and DAPI staining in invasive and noninvasive organoids. Scale bars: 50 μm. (**E**) Immunofluorescence images of pan-CK, SPOCK1, and DAPI staining in primary PDAC tissue sections. Scale bar: 50 μm.

A heatmap of the sample-to-sample distances in the RNA-seq data (Figure 2A) shows that invasive and noninvasive organoids from the same PDAC clustered most closely together, demonstrating shared transcriptomic features in organoids from the same tumor. However, a secondary pattern was observed where organoids from PDACs with the same invasive morphology also clustered together, suggesting some shared transcriptomic features based on invasive morphology in addition to patient-specific effects. The same pattern, with close clustering of organoids from the same PDAC but also clustering of organoids from PDACs with the same invasive morphology, was observed on a principal component analysis (PCA) plot of gene expression data from all profiled organoid samples (Supplemental Figure 1). To identify shared transcriptomes in collective and mesenchymal organoids, we performed differential gene expression analysis comparing the 12 samples of invasive organoids to the 11 samples of noninvasive organoids derived from the same primary tumors (Supplemental Table 4). Differential gene expression analysis identified 553 genes that were differentially expressed in invasive organoids (FDR < 0.05) (Figure 2B). Notably, among these 553 genes, 393 genes were upregulated in the invasive organoids, including *MMP8*, *OLR1*, *COL7A1*, and *SPOCK1*, all of which are implicated in extracellular matrix (ECM) structure and extracellular signaling (25–30).

We next investigated the impact of these differentially expressed genes on overall survival in a large independent cohort of PDAC samples. A Kaplan-Meier analysis (31) of The Cancer Genome Atlas (TCGA) PDAC cohort (3, 32) using the top 25 differentially expressed genes from our data set identified 11 genes with statistically significant inverse correlations with survival in upper and lower quartile expression groups (Supplemental Figure 2). *COL7A1* showed the most significant difference in survival in high and low expression groups, with a median survival time of 17 months in high *COL7A1* expressers compared with 38 months in low *COL7A1* expressers.

To confirm the protein expression of a subset of these differentially expressed genes in the PDAC organoids, we performed immunofluorescent labeling of the proteins encoded by *SPOCK1* and *OLR1* along with pan-cytokeratin (pan-CK) on representative collective, mesenchymal, and noninvasive organoids (Figure 2D, Supplemental Figure 3, and Supplemental Videos 1 and 2). SPOCK1 and OLR1 were chosen for assessment by immunofluorescence based on their significant differential expression in invasive organoids (Figure 2C and Supplemental Figure 3A), transmembrane and intracytoplasmic protein localization, and previously reported roles in cancer cell invasion. For example, *SPOCK1* (which encodes SPARC or osteonectin) is involved in cell-cell and cell-matrix interactions and has been associated with induction of EMT and invasion in multiple tumor types (28–30). In the noninvasive organoid, SPOCK1 showed uniform, moderate cytoplasmic staining. In contrast, SPOCK1 staining was more intense in the collective and mesenchymal organoids, with the strongest staining in the invasive protrusions facing the collagen I matrix. In addition, in the mesenchymal organoids, SPOCK1 staining highlighted single-cell projections from the core of the organoid. Immunofluorescence assessment of another differentially expressed gene, *OLR1*, showed a similar staining pattern, with protein expression enriched in the invasive protrusions of the collective and mesenchymal organoids (Supplemental Figure 3C and Supplemental Video 3 and 4).

We next sought to assess the expression of these proteins in cancer cells in primary PDAC tissue. To do this, we performed immunofluorescent staining to label SPOCK1 or OLR1, along with pan-CK immunofluorescence and DAPI staining, in formalin-fixed, paraffin-embedded (FFPE) tissue sections from a primary PDAC (Figure 2E and Supplemental Figure 3B). The PDAC cells showed a heterogeneous SPOCK1 or OLR1 staining intensity across the tissue section, but costaining with pan-CK confirmed the expression of both proteins in PDAC cells. Considering the complexity of PDAC architecture, the true “invasive front” of a tumor cannot be reliably identified in a 2-dimensional tissue section, so we could not determine whether SPOCK1 or OLR1 expression was enhanced in the most invasive PDAC cells in vivo. However, our results confirm the expression of SPOCK1 and OLR1 in PDAC cells in human tumors, and their heterogeneous expression is consistent with the patterns seen in our organoids.

**Figure 3:**
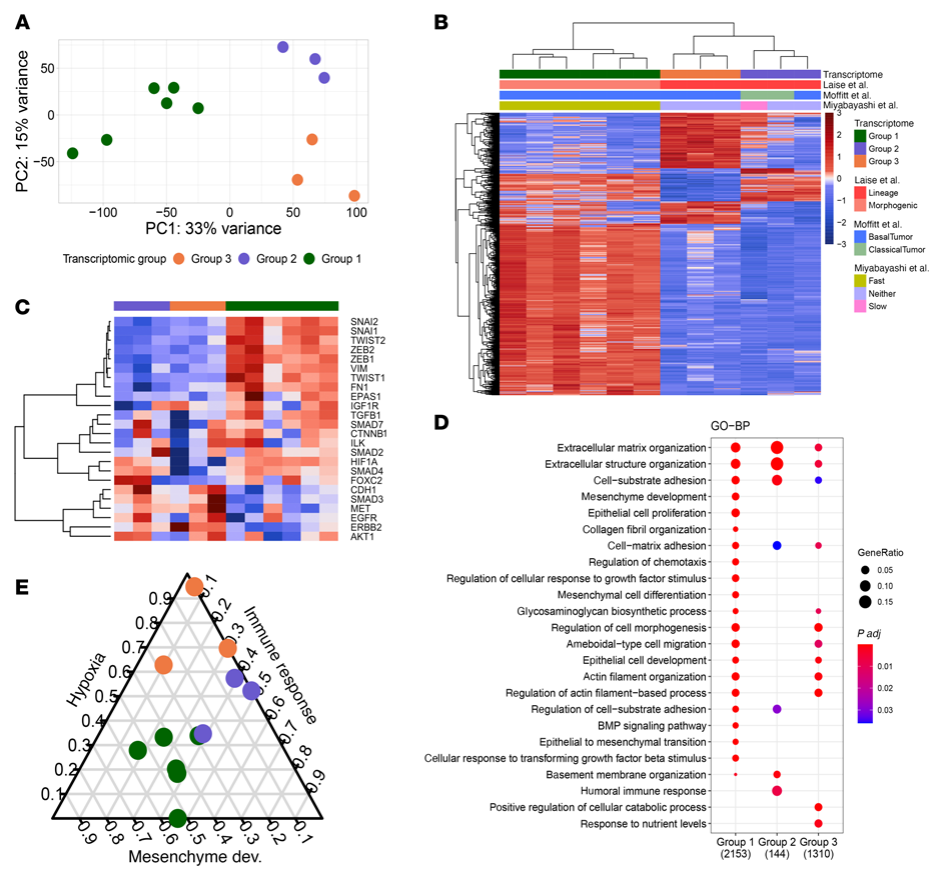
Transcriptomic analysis segregates invasive organoids into 3 distinct transcriptomic groups, each with unique pathway signatures. (**A**) PCA plot of gene expression in invasive organoids (*n* = 12). (**B**) Heatmap of differentially expressed genes between our 3 transcriptomic groups annotated by previously reported PDAC transcriptomic subtypes (8, 34) and phenotypic groups (35). (**C**) EMT pathway gene expression in all invasive organoids (*n* = 12). (**D**) Select pathways that are significantly enriched in differentially expressed genes between invasive (*n* = 12) and noninvasive counterparts (*n* = 11) from each of the 3 transcriptomic groups using Gene Ontology - Biological Processes. (**E**) Triangular plot using hypoxia, immune response, and mesenchymal development gene signatures on each axis.

To further examine the transcriptomic differences between invasive phenotypes, we limited our analysis to the 12 invasive organoid samples. PCA and non-negative matrix factorization (NMF) identified 3 distinct transcriptomic groups of invasive organoids (Figure 3, A and B). We found that on the transcriptomic level, the organoids do not simply separate into 2 groups based on their morphologic phenotype. While mesenchymal and collective organoids exclusively comprised transcriptomic groups 1 (tG1: 6 mesenchymal organoid cultures) and 2 (tG2: 3 collective organoid cultures), a third transcriptomic group emerged, which included both invasion patterns (tG3: 2 mesenchymal and 1 collective organoid cultures). These data suggest that although many of the molecular drivers of invasion are reflected in the invasive morphology, distinct molecular mechanisms that are not distinguishable morphologically (such as tG3) can be revealed by transcriptomic analysis.

We next sought to determine how our transcriptomic groups aligned with previously reported subtyping schemes for PDAC tissue. Using the DECODER pipeline (33) to infer associations with widely recognized classical versus basal transcriptomic subtyping scheme for primary PDAC tissue originally published by Moffitt et al. (8), 2 of 3 tG2 invasive organoid samples were assigned as the classical subtype, whereas the other invasive organoids were classified as the basal subtype (Figure 3B). Similarly, a recently proposed PDAC subtyping scheme using sets of transcriptional regulators (34) classified tG1 as being in the morphogenic state and both tG2 and tG3 as being in the lineage state. Lastly, we applied gene signatures previously derived from PDAC organoids distinguished by their speed of metastatic progression in murine intraductal xenografts (35). We found that the tG1 organoids expressed the “fast progressor” transcriptomic signature, whereas 1 collective organoid culture in tG2 expressed the “slow progressor” signature. However, the remaining invasive organoids expressed neither a slow nor fast progressor signature. Taken together, these analyses demonstrate that the transcriptomic groups identified in our organoid system correspond with transcriptomic subtyping previously reported in primary PDAC tissue samples, underscoring the fidelity of our organoid model and highlighting distinct invasion mechanisms as an important difference between transcriptomic subtypes.

A heatmap incorporating select EMT pathway genes (36, 37) demonstrated that the organoids in tG1 (6 mesenchymal organoid cultures) had the most prominently upregulated EMT transcriptional programs, with upregulated expression of *ZEB1/2*, *SNAIl1/2*, *TWIST1*, *VIM*, and *FN1* (Figure 3C). Conversely, organoids in tG2 and tG3 showed higher expression of *CDH1*, *SMAD3*, *MET*, *EGFR*, and *ERBB2* genes, which are associated with the TGF-β and phosphatidylinositol 3-kinase (PI3K) pathways. Interestingly, the organoids in tG3 exclusively showed lower expression of *HIF1A*, *SMAD4*, and *FOXC2*. These results show that unique gene modules within the EMT transcriptional programs are associated with distinct invasive phenotypes.

We next performed pathway analysis of Gene Ontology Biological Processes (GO-BP), using differentially expressed genes between invasive organoids and their noninvasive counterparts in each of the 3 transcriptomic groups (Figure 3D and Supplemental Table 5). The GO-BP enrichment revealed that all 3 transcriptomic groups of invasive organoids had differentially expressed genes related to ECM organization compared with noninvasive counterparts, which further highlights the importance of extracellular signaling in local invasion. tG1, which contained only mesenchymal organoids, showed the most robust enrichment for mesenchymal development (EMT and mesenchymal cell differentiation) as well as cancer-ECM interactions (cell-substrate adhesion, cell-matrix adhesion, cellular response to growth factor/TGF-β stimulus). tG2, which contained only collective organoids, yielded fewer differentially expressed genes than other groups but showed enrichment in regulation of cell-substrate adhesion, basement membrane organization, and humoral immune response. The tG3 invasive organoids were enriched for actin cytoskeleton organization and regulation of cell morphogenesis. This analysis demonstrates that while some pathways are upregulated in invasive organoids in all transcriptomic groups, each group also has distinct pathways that define its invasive organoids.

Based on unique pathway enrichment in each transcriptomic group, we projected all invasive organoid cultures on a triangular spectrum using hypoxia, mesenchyme development, and immune response gene expression as each axis (Figure 3E). Each axis, read in a clockwise manner, shows the relative fraction of that signature expressed in each sample. For example, a sample located in the top corner would only express the hypoxia-related genes and not the other 2 gene sets. A robust segregation of 3 transcriptomic groups was recapitulated by these 3 pathways, demonstrating the role of mesenchymal features, hypoxia, and the immune response in defining the distinct molecular features of each group. Furthermore, the triangular plot highlights the cell state continuum of our organoids with respect to these gene sets, as most of the samples are not located near a corner in the plot.

**Figure 4:**
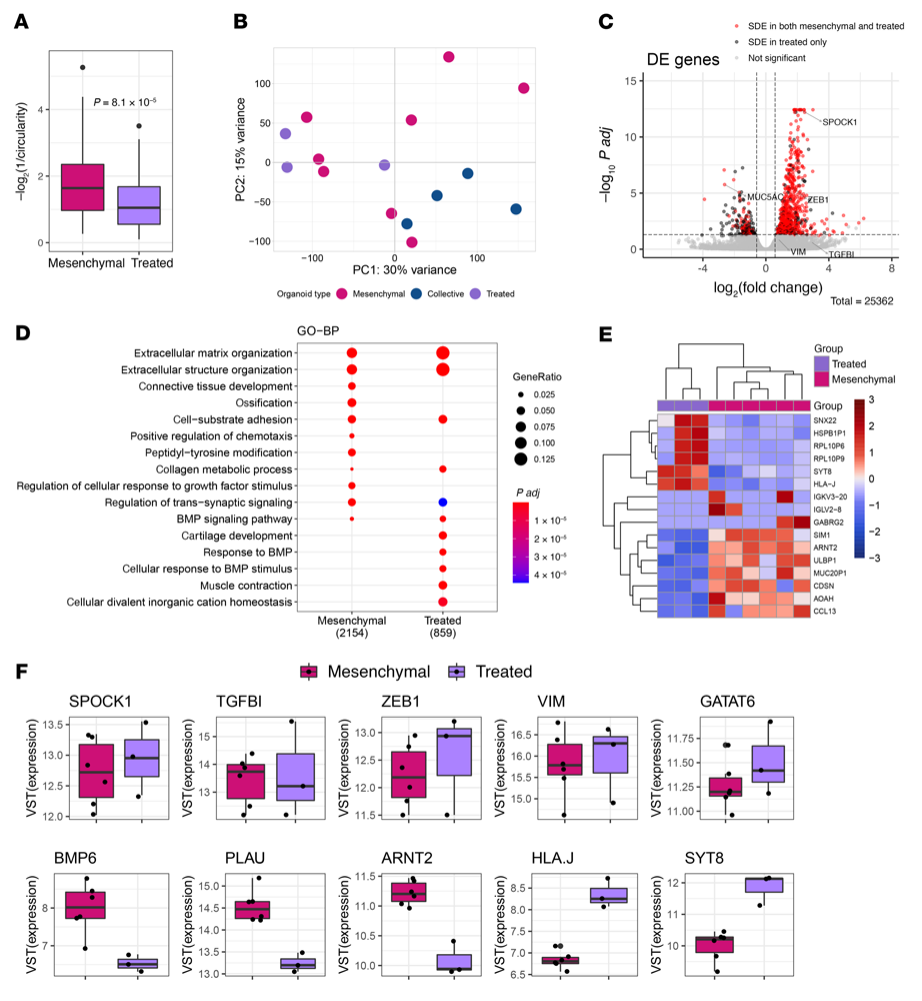
PDAC organoids from patients receiving neoadjuvant chemotherapy display similar invasion-associated transcriptional alterations to those of treatment-naive organoids. (**A**) Inverse circularity scores of mesenchymal organoid cultures generated from untreated (mesenchymal organoids, *n* = 8) and neoadjuvant-treated (treated, *n* = 3) PDACs (Wilcoxon’s test, *P* = 8.1 × 10^–5^). (**B**) PCA of gene expression in collective (*n* = 4), mesenchymal (*n* = 8), and treated organoids (*n* = 3). (**C**) Differentially expressed (DE) genes between neoadjuvant-treated invasive and noninvasive organoids (FWER 0.05) and overlap with DE genes between mesenchymal organoids and noninvasive counterparts. (**D**) Pathway analysis of mesenchymal and treated organoids using Gene Ontology - Biological Processes. (**E**) DE genes between treated and mesenchymal organoids. (**F**) mRNA expression levels of indicated genes in mesenchymal (*n* = 8) and treated organoids (*n* = 3). Each dot represents an organoid culture. In the box-and-whisker plots, the box outlines represent the 25th to 75th percentiles, horizontal lines represent medians, and whiskers extend to 1.5 × IQR.

Neoadjuvant chemotherapy is increasingly employed in the clinical care of PDAC patients, providing an important opportunity to study the impact of chemotherapy on invasion in organoids derived from surgically resected PDACs. Therefore, we next sought to assess invasion-associated transcriptomic alterations in human PDAC organoids from patients who underwent neoadjuvant chemotherapy. Using the same organoid dissection and RNA-seq pipeline, we analyzed organoids from 3 patients who were treated with neoadjuvant chemotherapy (FOLFIRINOX alone or FOLFIRINOX with gemcitabine/Abraxane) prior to surgery (Supplemental Table 1). Organoids from all 3 PDACs invaded with a mesenchymal phenotype, but these treated organoids had lower inverse circularity (*P* < 0.001) and lower mean invasive protrusions (3.6 vs. 5.2, *P* = 0.012 by unpaired *t* test) than organoids from treatment-naive PDACs (Figure 4A).

PCA analysis showed the transcriptomes of the invasive organoid generated from neoadjuvant-treated tumors clustered with the tG1 organoids (untreated mesenchymal organoids) (Figure 4B). A differential gene expression analysis comparing the invasive and noninvasive organoids from chemotherapy-treated tumors yielded 859 differentially expressed genes (FDR < 0.05) (Figure 4, C and D, and Supplemental Table 6). As expected, a majority of these differentially expressed genes overlapped with differentially expressed genes from comparing untreated tG1 organoids to noninvasive counterparts (Figure 4C). Furthermore, we identified 16 differentially expressed genes from comparing neoadjuvant therapy–treated invasive organoids to untreated tG1 mesenchymal organoids (*P* < 2 × 10^–6^) (Figure 4E). Among these genes, 6 genes, including *SYT8* and *HLAJ*, were upregulated in the chemotherapy-treated organoids. We show the variance-stabilizing transform of the expression of select EMT related genes (*SPOCK1*, *TGFBI*, *ZEB1*, *VIM*) and classical subtype gene (*GATA6*) that were not different between chemotherapy-treated and tG1 organoids as well as select genes that were downregulated (*BM6*, *ARNT2*, *PLAU*) and upregulated (*SYT8*, *HLAJ*) in the chemotherapy-treated organoids (Figure 4F). Some of these have been associated with chemotherapy resistance in other tumor types (38–41).

We next sought to examine whether the transcriptomic groups identified in our organoid data could be translated to other publicly available transcriptomic data from PDAC tissue.

**Figure 5:**
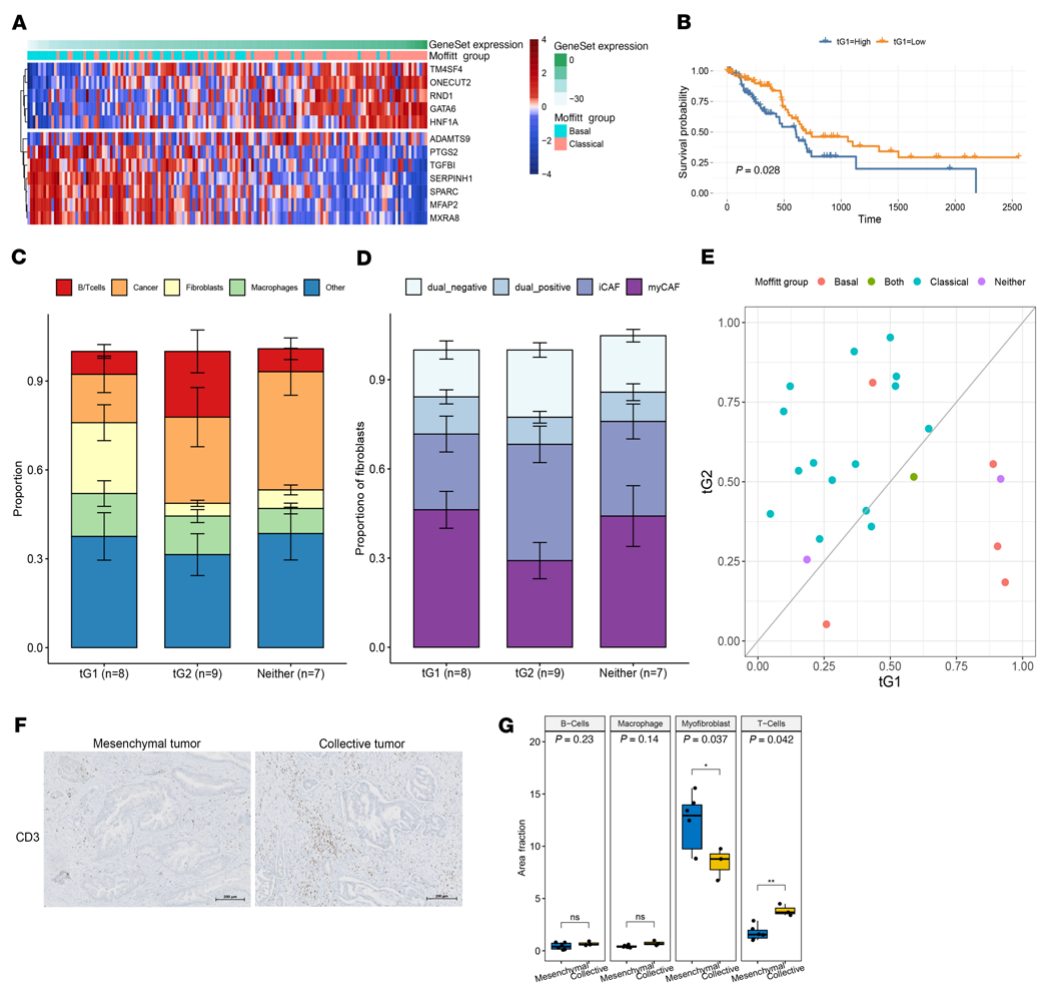
Application of organoid invasion signatures to primary PDAC scRNA-seq data reveals different cellular compositions in the tumor microenvironment. (**A**) Pancreatic cancer samples from The Cancer Genome Atlas (PAAD from TCGA) ordered by their expression score of tG1 and tG2 genes (the score was calculated using Ucell R package of positive expression of tG2 genes and negative expression of tG1 genes). This gene expression score is annotated in green at the top of the figure. The second annotation bar shows the Moffitt et al. (8) basal and classical subtypes for these samples. (**B**) A Kaplan-Meier curve of high and low tG1 signature–expressing tumors in TCGA cohort (*n* = 185). (**C**) Average fractions and error bars showing SD of neoplastic cells, T and B cells, fibroblasts, macrophages, and other cell types within tG1 (*n* = 8), tG2 (*n* = 9), and neither (*n* = 7) tumors in the Peng et al. (42) scRNA-seq data set. (**D**) Average fractions and error bars showing SD of fibroblast subpopulations in tG1 (*n* = 8), tG2 (*n* = 9), and neither (*n* = 7) tumors from Peng et al. scRNA-seq data set. (**E**) Fraction of tumor cells that express tG1 genes (*x* axis) and tG2 genes(*y* axis) within each PDAC in the Peng et al. scRNA-seq data set. Each patient’s Moffitt et al. subtype is shown in the color legend. (**F**) Representative images of immunohistochemical staining showing the density of CD3^+^ T cells in primary tumors that produce mesenchymal and collective organoids. Scale bars: 200 μm. (**G**) Quantification of the density of B cells, T cells, myofibroblasts, and macrophages/tumor area (100 μm^2^) on tissue sections of primary PDACs (6 tumors that generated mesenchymal organoids and 3 that generated the collective organoids), which shows statistically significant differences in fractions of myofibroblasts (*P* = 0.037, *t* test) and T cells (*P* = 0.0042, *t* test). Each dot represents 1 tumor. In the box-and-whisker plots, the box outlines represent the 25th to 75th percentiles, horizontal lines represent medians, and whiskers extend to 1.5 × IQR.

First, we derived a minimal set of genes for tG1 and tG2 that belong to unique phenotypes (mesenchymal and collective, respectively) and applied them to the 185 PDAC samples available in TCGA (Figure 5A) (3, 32). We found that by separating the patients into 2 groups based on their mean expression of these genes, the patients with high expression of genes from tG1 had a significantly lower overall survival rate relative to the low tG1 group (Figure 5B). Next, we leveraged publicly available human PDAC scRNA-seq data (*n* = 24) with provided cell-type annotations (42), which allowed us to assess correlation of our transcriptomic groups with features of the tumor microenvironment. By applying gene sets of tG1 (6 mesenchymal organoid cultures) and tG2 (3 collective organoid cultures) to the cancer cells in the human PDAC scRNA-seq data set, we confirmed that both tG1 and tG2 signatures are present in the PDAC scRNA-seq data. Tumors with PDAC cells showing the highest fraction of tG1 or tG2 signatures were designated as tG1 (*n* = 8) or tG2 (*n* = 9) groups. In addition, we also found some tumors that contained neither transcriptomic group (*n* = 7). In these groups, we identified significant differences in the proportion of cancer cells and non-neoplastic cells within the tumor microenvironment (Figure 5C). Tumors with neoplastic cells expressing the tG2 signature had a significantly higher proportion of neoplastic cells and T and B cells in the tumor microenvironment when compared with the tG1 group tumors. Moreover, the microenvironment of the tG1 tumors had a higher proportion of fibroblasts than the tG2 and neither groups. Lastly, tumors that express neither the tG1 or tG2 signatures had the largest fraction of neoplastic cells and the least immune infiltration in the tumor microenvironment.

Next, we compared the fibroblast subpopulations to further examine the distinct tumor microenvironment compositions of the tG1, tG2, and neither-group tumors from the PDAC scRNA-seq data set. Intriguingly, we observed differences in inflammatory and myofibroblastic cancer-associated fibroblast (iCAF and myCAF) enrichment between these groups of tumors. While the tG1 and neither-group tumors showed higher proportions of fibroblasts expressing the myCAF signature (43), the tG2 tumors’ fibroblasts consisted of higher fractions of iCAFs (Figure 5D). We next compared our transcriptomic groups in this scRNA-seq data set to the Moffitt et al. subtyping scheme (8). We found that patients with majority classical cells had a higher fraction of cells with tG2-related genes and the patients with majority basal cells had higher fractions of cells that express the tG1 genes (Figure 5E). These observations showed again, in an independent data set, that our transcriptomic groups from the organoid model align well with the Moffitt subtypes (as in Figure 3B).

To validate our findings, we performed immunohistochemical labeling of T cells (CD3), B cells (CD20), myofibroblasts (αSMA), and macrophages (CD68) on sections of primary tumor tissue from our original cohort (Figure 5F). For each cell type, we calculated the density of the cells/tumor area (100 μm^2^) (Figure 5G). The PDACs that gave rise to collective organoids showed significantly higher density of T cells than the tumors that produced mesenchymal organoids (*P* = 0.0042, *t* test). Conversely, PDACs that gave rise to mesenchymal organoids had higher density of myofibroblasts (*P* = 0.037, *t* test). These patterns are consistent with our predictions based on the application of our transcriptomics signatures to the independent scRNA-seq data set (Figure 5, C and D).

**Figure 6:**
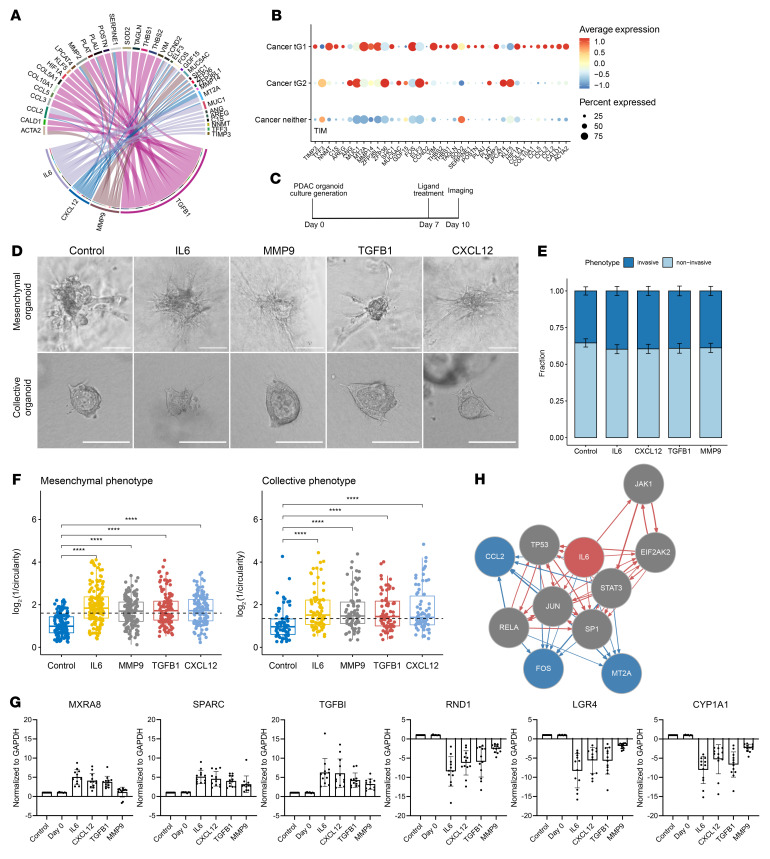
Identification and experimental evaluation of distinct ligand-receptor interactions between fibroblasts and tumor cells belonging to tG1. (**A**) A circos plot showing the 4 ligands that were tested experimentally and their target genes. (**B**) List of fibroblast ligand target genes and activation status in tG1 (*n* = 8), tG2 (*n* = 9), and neither (*n* = 7) group tumors. (**C**) Timeline of ligand treatment assay. (**D**) Representative images of the control, IL-6–, MMP9-, TGF-β1–, and CXCL12-treated collective and mesenchymal organoids after the ligand treatment assays. Scale bars: 50 μm. (**E**) Proportion of invasive (dark blue) and noninvasive (light blue) organoids per ligand condition (*n* = 12). (**F**) A metric of organoid invasive morphology (log_2_[1/circularity]) for control and ligand-treated mesenchymal (*n* = 9) and collective organoid cultures (*n* = 4). ****P ≤ 0.0001 by Mann-Whitney *U* test. Each dot represents a PDAC organoid; data are shown as box-and-whisker plots, with the box boundaries representing the 25th to 75th percentiles, horizontal lines representing medians, and whiskers that extend to 1.5 × IQR. Dashed line shows average shape metric across all 4 treatment groups. (**G**) qRT-PCR showing upregulation in mRNA levels of tG1 genes (*MXRA8*, *SPARC*, *TGFBI*) and downregulation in mRNA levels of tG2 genes (*RND1*, *LGR4*, *CYP1A1*) after ligand treatment assay (presented as mean ± SD for 12 patients). Day 0 samples and untreated controls showed no significant differences in mRNA expression levels of the genes (Student’s *t* test, *P* > 0.05); however, the differences between the control and all treated organoids were significant (Student’s *t* test, *P* < 0.01), except for the *MXRA8* gene in MMP9-treated cultures (Student’s *t* test, *P* > 0.05). (**H**) A prediction of downstream genes in the IL-6 pathway

In order to identify ligands from fibroblasts that could be driving the transcriptomic signature in tG1, we conducted ligand-target gene pairing analysis using NicheNet (44). We focused on fibroblasts in tG1 tumors because the tG1 group had the highest proportion of fibroblasts in our analysis of scRNA-seq data, suggesting that fibroblasts could be a microenvironmental driver of invasion in tG1 tumors. For this ligand-target analysis, we used patient samples from the scRNA-seq data set that had high tG1 signatures. We used genes that were enriched in the fibroblasts from these patients as potential ligands. For potential target genes, we used genes that were enriched in the cancer cells from these patients compared with the patients in the “neither” group (Figure 6A). We further restricted our results to genes that were differentially expressed in tG1 mesenchymal organoids compared with their noninvasive counterparts. The analysis yielded several interesting ligand-target gene pairs, including ligands with previously reported roles in tumor cell invasion (Figure 6B). In order to validate a subset of these ligands, we tested the impact of TGF-β1, IL-6, CXCL12, and MMP9 on invasion in 12 freshly derived human PDAC organoids cultures in collagen I gels (Figure 6, C and D), including 8 cultures with predominantly mesenchymal invasion, 3 cultures with predominantly collective invasion, and 1 culture containing both phenotypes. The proportion of the invasive and noninvasive organoids was similar between the control and the treatment groups (Figure 6E). However, the inverse circularity scores of TGF-β1–, IL-6–, CXCL12-, and MMP9-treated organoids were significantly higher than the controls in all analyzed PDAC organoid cultures (Figure 6F and Supplemental Figure 4). Of note, the invasive phenotypes were not altered by ligand treatment; cultures with predominantly collective invasion in control conditions retained this phenotype with ligand treatment (Supplemental Videos 5 and 6). To determine whether ligand treatment altered the expression of genes associated with our transcriptomic groups, we performed quantitative reverse transcriptase PCR (qRT-PCR) of a panel of 3 genes from tG1 and 3 genes from tG2 on all ligand-treated organoid cultures from these 12 patients. Treatment with all 4 ligands led to upregulation of tG1 genes and downregulation of tG2 genes (Figure 6G), supporting the hypothesis that ligands from fibroblasts enhance invasion via the tG1 transcriptomic program. Intriguingly, the upregulation of tG1 genes and downregulation of tG2 genes occurred in both mesenchymal and collective organoid cultures, demonstrating that upregulation of tG1 genes can also enhance collective invasion. Taken together, these results validate that TGF-β1, IL-6, CXCL12, and MMP9 enhance invasion in PDAC organoids, and the enhancement of invasion by these ligands (which were identified through analysis of tG1 samples) is not limited to a specific invasive phenotype. Among these ligands, IL-6 treatment resulted in the strongest enhancement of the invasive phenotype, as evidenced by the highest increase in the inverse circularity score. Some of the downstream genes of the IL-6 pathway are illustrated in Figure 6H and include *TP53*, *JAK*, *STAT3*, and *JUN*.

## Discussion

In this study, we leveraged our organoid culture system of human PDAC samples, employing a unique dissection approach after culture in collagen I gels, to isolate organoids for molecular profiling based on their invasive phenotype. From each organoid sample, we separately analyzed invasive and noninvasive organoids, and the invasive organoids were further classified as mesenchymal or collective, with a predominant phenotype in each patient sample. The results of the organoid transcriptomic profiling provide important insights into the molecular programs that underlie invasion. We identified significantly differentially expressed genes, including *COL7A1*, *SPOCK1*, and *OLR1*, when comparing invasive organoids with noninvasive organoids from all patients (Figure 2). These genes, which were associated with worse prognosis in an independent cohort, suggest a conserved molecular program that drives invasion.

In addition, clustering of the transcriptomes of the invasive organoids from each patient revealed 3 molecularly distinct groups, suggesting some heterogeneity in the molecular invasion programs between patients. Two of these groups contained only organoids of a single morphological invasive phenotype, confirming that these distinct phenotypes leverage different molecular programs to invade. However, the third group was mixed in phenotype. This indicates that our phenotypic classification of organoid invasion captures molecularly distinct groups, but all of the molecular differences are not evident morphologically. Although our transcriptomic groups were derived using whole transcriptome data, we were able to recreate the groups using only pathways related to mesenchymal development, hypoxia, and immune response, highlighting that these are among the key molecular pathways in the distinct mechanisms of PDAC invasion. In addition, our results underscore the heterogeneity of invasive potential of cancer cells within each patient. Every tumor sample produced both invasive and noninvasive organoids, demonstrating that all PDAC cells within a given patient are not equally invasive.

Multiple previous studies have performed transcriptomic profiling of primary human PDAC tumors, producing several proposed classification schemes (10–13). Recent integration of these data has delineated 2 consensus transcriptomic groups, typically termed classical and basal, with basal tumors having a worse prognosis (8). Our organoid transcriptomic groups overlap substantially with the gene signatures for the classical and basal groups (Figure 3). This concordance suggests that our signatures derived from RNA-seq of organoid cultures are relevant to human PDACs in vivo. In addition, it suggests functional and molecular differences in the programs of invasion in these previously defined subgroups. This functional difference is also highlighted in the overlap of our groups from the signatures of “fast progressors” and “slow progressors” identified by intraductal injection of human PDAC organoids into the murine pancreas (35), suggesting that the fast and slow progressors are not only molecularly different but employ phenotypically distinct invasion patterns. The correlation of our tG1 signature with decreased overall survival is in line with associations of the basal and fast progressor subtypes (8), as well as our previous observation of worse prognosis in tumors giving rise to organoids with mesenchymal phenotype (21).

In addition to the 2 transcriptomic groups that discreetly contain mesenchymal and collective invaders, we identified a third transcriptomic group containing organoids of both phenotypes. The existence of a third “intermediate” cell state has been proposed by recent bulk RNA-seq and scRNA-seq studies of human PDAC samples (11, 45). Moreover, multiple studies have reported cells with “biphenotypic” epithelial and mesenchymal features (sometimes referred to as partial EMT), underscoring the possibility for transitions between these states (46–48). However, while some transcriptomic features of tG3 align with intermediate cell states in other studies (49), several features of our data set suggest that tG3 is not simply a transition or mixture of tG1 and tG2. In the 3-way transcriptomic comparison of the invasive organoids in our data set (Figure 3B), there was a set of genes that were uniquely upregulated in tG3 and not seen in tG1 and tG2, and tG3 also had the highest expression of genes in the hypoxia gene signature (Figure 3E), suggesting that tG3 is a distinct rather than an intermediate transcriptomic group. Single-cell or spatially resolved transcriptomic approaches in organoid models will be required in future studies to more robustly address this question.

When we applied these organoid-derived transcriptomic signatures to scRNA-seq data from human primary PDACs, we found significant differences in the non-neoplastic cells in the tumor microenvironment between PDACs with different predominant signatures. The suggested impact of the tumor microenvironment on invasion is supported by our previous studies in human PDAC organoids, which demonstrated attenuated invasion with passaging in epithelium-only culture (21). However, the differences in microenvironment between tumors with tG1 and tG2 signatures suggest that non-neoplastic cells in the surrounding microenvironment may impact not only the extent of invasion but also underlying molecular features. Using ligand-receptor analysis, we identified several ligands from fibroblasts with targets overexpressed in mesenchymal invaders in tG1. These included multiple ligands with previously supported roles in cancer cell invasion, including TGF-β1, IL-6, CXCL12, and MMP9, underscoring the robustness of our approach. For example, we previously showed that TGF-β treatment can enhance invasion in some PDAC organoids (21), and previous PDAC-CAF coculture studies identified TGF-β1 secreted from CAFs as a key mediator of tumor cell proliferation and EMT (50). In addition, multiple studies have demonstrated that IL-6 can enhance migration of PDAC cell lines in 2-dimensional culture (51, 52). Other ligands, such as CXCL12 and MMP9, have documented roles in invasion in other tumor types, but their role in PDAC invasion remains to be explored in depth (53, 54). We experimentally validated several of these ligands in our PDAC organoid system, confirming their ability to enhance invasion in human PDAC cells. We also showed that while these ligands can alter the invasion-associated gene signatures, single ligand treatment does not shift the morphologically defined invasive phenotype (collective or mesenchymal), at least in the time course of our validation experiments. Taken in the context of our previous work on PDAC organoids (21), these results suggest that the invasive phenotype is likely driven by a combination of molecular alterations in tumor cells and alterations in cellular composition of the tumor microenvironment.

Previous studies have reported utilization of human PDAC organoids for a range of organoid profiling questions, including drug response (55, 56) and growth factor dependency (57). Our approach employs human PDAC organoids grown in collagen I gels to interrogate the molecular programs underlying invasion. In this study, we analyzed freshly derived organoids from surgically resected human PDACs; the use of fresh organoids to assess transcriptomic programs is important, as recent reports have shown a transcriptomic shift in human PDAC organoids after long-term passaging in culture (45). However, the use of fresh organoids without expansion over passages in culture limits the quantity of organoids available for analysis, and some assays, such as murine implantation (35) or large-scale drug screening (55, 58), may not be possible with this approach in PDAC. Another important caveat is that our study included only organoids from surgically resected PDACs, which represent the minority of newly diagnosed PDAC patients (1, 2). Others have recently demonstrated the ability to derive organoids from small biopsy material (58, 59), opening the possibility to derive organoids from PDAC metastases. Assessment of these invasive phenotypes and transcriptomic programs in organoids across the PDAC stage spectrum is an important future direction. In addition, in order to better understand the native biology of invasion of PDAC cells, we focused mostly on PDACs that did not receive neoadjuvant chemotherapy. Our RNA-seq results on a limited number of treated PDACs suggest that they maintain similar invasion-associated transcriptomic programs. In addition, we previously reported a similar prevalence of invasive phenotypes in organoids derived from surgically resected PDACs with and without neoadjuvant chemotherapy (21), and in the current study, these prevalences are similar in our initial transcriptomic analysis cohort (untreated) and ligand-validation cohort (treated), providing further evidence that neoadjuvant therapy does not alter invasive phenotype. Still, direct comparisons of pre- and posttreatment samples from the same patient will be required to fully understand the impact of chemotherapy on invasion and its associated molecular alterations.

Our study leverages a sizable cohort of human PDAC organoids processed through our organoid dissection and transcriptomic analysis pipeline in order to delineate the molecular programs that drive invasion. We identify transcriptomic groups that align with morphologically defined organoid invasion patterns, and through correlation with primary PDAC scRNA-seq data, we identify specific ligands from non-neoplastic cells that enhance invasion in our organoid model. Taken together, our results provide important insights into the molecular and cellular alterations that drive invasion in human pancreatic cancer, highlighting potential strategies to inhibit invasion for novel therapeutic approaches.

## Methods

### Human pancreatic cancer specimens.

Fresh tissue was harvested from 14 surgically resected human pancreatic cancer specimens at the time of gross examination in Surgical Pathology at the Johns Hopkins University Hospital or the University of Mainz Medical Center.

### Organoid generation.

Organoids were generated from resected PDAC and pancreaticobiliary ampullary carcinoma as described in the literature (21). Fresh PDAC specimens were digested within 24 hours of surgical resection. Organoids were rinsed with a solution containing 10,000 U/mL penicillin, 10 mg/mL streptomycin (Sigma-Aldrich, P4333), and amphotericin B (Invitrogen, 15290026) in DPBS (Sigma-Aldrich, D8537). Tumors were minced and shaken in digestion solution containing 1 mg/mL collagenase from *Clostridium* *histolyticum* (Sigma-Aldrich, C2139), insulin (Sigma-Aldrich, I9278), penicillin-streptomycin (Sigma-Aldrich, P4333), L-glutamine (Gibco, 25030081), and 5% FBS (Gibco, A4766801) in DMEM (Sigma-Aldrich, D6546) up to 2 hours, depending on the size of the tumor.

The cancer cell suspension was centrifuged for 10 minutes at 1500 RPM at 4°C. The digestion media was aspirated, and 4 mL of DMEM/F-12 (Sigma-Aldrich, D6421) was added, and 40 μL of DNase (Sigma-Aldrich, D4263) was added and shaken for 3 minutes to remove any fragmented DNA in the digestion solution. The cancer cell suspension was centrifuged for 10 minutes at 1500 rpm at 4°C, and the pellet was washed with 10 mL of DMEM/F-12. The cancer cell suspension was placed on a tube rack to allow large stromal pieces to precipitate. The supernatant containing organoid and single-cell suspensions was moved to a new tube to repeat 5 seconds of centrifugation and washing 4 times with DMEM/F-12. In general, 20 μL of cell suspension contained 20–30 organoids, and this number was checked under a microscope to plate 50–60 organoids per dome. Collagen I gel was prepared as previously described (21, 55). Collagen I was mixed with 10× DMEM and 1N NaOH to result in 3.34 mg/mL collagen (Corning, collagen I, rat, 354236). The collagen solution was incubated in ice for 30 minutes until collagen fiber formation was initiated. The collagen gel was then mixed with an organoid pellet and plated on a 60-mm dish (Corning, 430166) in a flattened dome shape and was solidified at 37°C for 30 minutes. After the collagen I gel solidified, the pancreatic organoid media containing insulin (Sigma-Aldrich, I9278), GlutaMAX (Gibco, 35050061), Pen-Strep (Sigma-Aldrich, P4333), 0.0075% BSA (Sigma-Aldrich, A9647), cholera toxin (Sigma-Aldrich, C8052), and human EGF (Sigma-Aldrich, E9644) in DMEM (Sigma-Aldrich, D6546) was added.

### Microscopy.

Representative images of invasive and noninvasive organoids were acquired using a Nikon Ti-E inverted microscope on day 5 or 6 of culture, 1–2 days prior to collection. All images were taken at ×10 magnification.

### Organoid collection.

The manual collection of individual organoids was performed on an inverted microscope (Nikon TMS). Prior to collection, the pancreatic organoid medium was aspirated and replaced with 2 mL of PBS to prevent the collagen I gel from drying. A pair of extra-fine forceps (Excelta 5-SA-SE Tweezer), each designated for the invasive or noninvasive phenotype, was used. Briefly, the organoid of interest was identified, and many small holes were made in the collagen gel around it. Then, the collagen I gel surrounding the organoid of interest was gently grasped by the forceps and transferred to a tube. After the collection, 1 mL of TRIzol (Invitrogen, 15596026) was added to each tube, and the tubes stored at –80°C until RNA extraction.

### Immunofluorescent staining.

Whole domes containing organoids were fixed with 4% paraformaldehyde overnight (Electron Microscopy Sciences, 157-4) and stored in DPBS (Sigma-Aldrich, D8537) until imaging. Organoids were permeabilized with permeabilization buffer containing 0.5% Triton X-100 (Sigma-Aldrich, T9284) in DPBS. Blocking buffer (10% FBS, 1% BSA, 0.1% Triton X-100 in DPBS) was used for blocking nonspecific sites. Primary antibodies against pan-CK (Cell Signaling Technology, 4545S), OLR1 (Invitrogen, PA5-80872), SPOCK1 (Abcam, ab229935), and COL7A1 (Invitrogen, PA5-139764) were diluted at 1:400 (pan-CK and OLR1), 1:300 (SPOCK1), and 1:25 (COL7A1) in antibody dilution buffer (1% FBS, 1% BSA, 0.1% Triton X-100 in DPBS) and incubated on a tilting shaker overnight at 4°C. The next day, organoids were washed with DPBS for 10 minutes on a shaker 3 times. Next, secondary antibodies (Invitrogen, A31572 and A11001) in antibody dilution buffer were incubated 3 hours. Finally, DAPI (Invitrogen D21490) in dilution buffer was added and incubated for 10 minutes before imaging. All dilution buffer and DPBS were aspirated prior to imaging.

The immunofluorescence protocol for PDAC FFPE sections was the same except for the additional antigen retrieval process. Briefly, the FFPE sections were immersed in xylene to remove paraffin and washed by dipping in serial ethanol and deionized water. Lastly, the FFPE sections were incubated in sodium citrate buffer (pH 6.0) at 100°C. Finally, the stained slides were coverslipped. All images were acquired using a Nikon A-1 Confocal microscope and Nikon Ti-E inverted microscope, and image processing was performed using ImageJ (60).

### RNA-seq library generation.

Library preparation and RNA-seq were performed at the Single Cell & Transcriptomics Core at Johns Hopkins using the TruSeq stranded RNA-seq library kit (Illumina). Libraries were prepared using 30–100 ng of RNA from each culture. For every invasive and noninvasive pair, the starting RNA input was matched for library preparation. The libraries were sequenced on the Illumina Next-Seq and Nova-Seq platforms, with coverage of 40–60 million reads per sample. The quality of the reads was evaluated using FASTQC software (61).

### Targeted DNA sequencing analysis.

Targeted NGS sequencing was performed using the Solid Tumor gene list v4, which includes 432 cancer hotspot genes at Johns Hopkins Genomics. DNA was extracted from FFPE tissue specimens, captured with Kapa Roche reagents and Integrated DNA Technology (IDT) probes, and sequenced using the Illumina Hi-Seq and Nova-Seq platforms. Analysis was performed using human reference sequence genome assembly hg19 (GRCh37). Variant callers (MDL VC 9.0 and Haplotyper Genome Analysis TK-3.3) using the Bayesian statistical model were used to generate a list of variants. Multiple annotations and filtering algorithms, including the COSMIC database v88 (62), dbSNP v150 (63), and Annovar (64), were used to confirm mutation status. Furthermore, DNA mutations with variant allele frequency greater than or equal to 0.25 were filtered out to exclude germline mutations. Copy number alterations were called when the log_2_(mean chromosomal copy number) was greater than or equal to 1.3 (amplification) or less than or equal to –1.0 (loss). These annotations were reviewed by a team of laboratory technologists, genetic analysts, and pathologists.

### RNA-seq analysis.

The human genome was obtained in FASTA format (GRCh38) from Ensembl (65) and gene set annotation in GTF format. The hisat2 indices were built from the genome index using hisat2-build from Hisat2 version 2.1.0 (66). Raw RNA-seq paired-end reads were aligned to the genome using hisat2 after trimming with TrimGalore (67). The total reads per sample ranged from 37–62 million and the alignment mapping rate was greater than 90%. We next used DESeq2 (68) to estimate differential gene expression between invasive and noninvasive organoids in each invasion mode from the counts generated by HTSeq (69). We used standard DESeq2 parameters to exclude genes with no reads and those with *P* values set to the nominal value of 1. Additionally, we removed genes with low read count by requiring an average of 5 reads per patient sample.

### Pathway analysis.

We performed pathway analysis using the R package clusterProfiler (70) for KEGG (71) and GO (72, 73) and our own algorithm for gene set enrichment analysis of the MSigDB pathways from the Broad Institute (74). The triangular plot in Figure 3E was created using the R package Plotrix (75) using the triax.plot function and the pathways used were cancer hypoxia (76), mesenchyme development (72, 73), and immune response (8).

### NMF analysis.

We performed NMF (77) on all 12 cultures with organoids that invaded the collagen I matrix. We used variance-stabilizing transformation of the count data and identified 3 distinct groups in the analysis (parameters nrun = 250 and others set to default). We extracted the feature genes for each of the 3 groups and cross-referenced them with genes that were differentially expressed between invasive organoids in each NMF group relative to their noninvasive counterparts.

### scRNA-seq analyses.

We used publicly available scRNA-seq data for 24 primary PDAC tumors (42). These data were selected due to their large size (39,646 cells from 24 PDAC patients) and high number of CAFs (5,823 cells) relative to other currently available data sets. Cell-type annotations, including subtypes based on established phenotypes for epithelial cancer cells (basal-like and classical gene expression programs) (8) and CAFs (myofibroblastic and inflammatory properties) (78) were obtained. Briefly, the Seurat package (v4.0.1) (79, 80) for R statistics (v4.0) was utilized to calculate gene set scores for cells within the epithelial cancer or CAF populations. Cancer cells with a positive basal-like score and negative classical score were classified as basal, whereas cancer cells with a negative basal-like score and positive classical score were defined as classical (otherwise dual-positive or dual-negative). Fibroblasts with a positive myofibroblastic score and negative inflammatory score were classified as myCAFs, whereas fibroblasts with a negative myofibroblastic score and positive inflammatory score were defined as iCAFs (otherwise dual-positive or dual-negative). Exploration and validation of the organoid-derived transcriptomic programs tG1 and tG2 was conducted by calculating the *U* score of their signature genes in the cancer cells. Patients were classified in either group if at least 20% of their cancer cells expressed that signature. After splitting the patients into tG1, tG2, or neither-expression groups, we quantified the combination of other cell types within their tumors.

### Ligand-target gene analysis.

To identify ligands on fibroblasts that could be responsible for gene expression changes observed on the cancer cells, we used the R package NicheNet (44) to infer ligand-target gene pairs. This algorithm incorporates intracellular signaling and transcriptional regulation to go beyond ligand-target pairing. We used the nichenet_seuratobj_cluster_de function, with the sender cell being fibroblasts from patients that belong to the tG1 group and the receiver cell being differentially expressed in the cancer cells belonging to the tG1 group relative to other cancer cells.

### Immunohistochemistry and analysis.

Antibodies against CD3 (DAKO, A045201-2; 1:100), CD20 (Roche/Ventana, 760-2531; prediluted), and CD68 (Roche/Ventana, 790-2931; prediluted) were used to label T cells, B cells, and macrophages on sections of primary tumors. For analysis, stained slides were scanned and HALO software v3.5 (Indica Labs) was used to annotate the tumor regions on the given sections. Multiple parameters, including a range of staining positivity threshold and nuclear and cell sizes representing each given cell type (T cell, B cell, macrophage) were manually set prior to analysis.

### Ligand treatment assay.

For the ligand treatment assay, freshly derived PDAC organoids were divided into 5 different wells on a 24-well plate and the following ligands were given at indicated concentrations in the feeding media: IL-6 (Peprotech, 200-06; 100 ng/mL), MMP9 (Sigma-Aldrich, PF024; 10 ng/mL), SDF-1 (CXCL12) (Peprotech, 300-28B; 100 ng/mL), and TGF-β1 (Biotechne, 7754-BH-005/CF; 5 ng/mL). The control organoids were given feeding media only. All PDAC organoids were imaged 72 hours after the ligand treatment.

### qRT-PCR.

Total RNA was extracted from the organoids in each dome using a Quick-RNA Miniprep Plus kit (Zymo Research, R1057) with DNase I treatment, following the manufacturer’s instructions. Isolated RNAs were directly reverse transcribed to cDNA with the High-Capacity RNA-to-cDNA Kit (Applied Biosystems, 4387406), as per the manufacturer’s instructions. Thereafter, using the TaqMan Universal Master Mix II (Applied Biosystems, 4440038), qRT-PCR was carried out with 3 separate experimental replicates for each of the following genes: *TGFBI* (IDT, Hs.PT.58.40018323), *SPARC* (IDT, Hs.PT.58.24878442), *MXRA8* (IDT, Hs.PT.58.27513130.gs), *LGR4* (IDT, Hs.PT.58.22915883), *RND1* (IDT, Hs.PT.58.26229791), and *CYP1A1* (IDT, Hs.PT.58.219047). The relative mRNA expression levels were calculated using the comparative threshold cycle (dCt) method and normalized to *GAPDH* as the reference gene (IDT, Hs.PT.39a.22214836).

### Statistics.

Statistical analyses, including the Mann-Whitney *U* test, χ^2^ test, and 2-tailed Student’s *t* test comparing the clinical and pathological features of tumors and organoid inverse circularity scores were conducted using SPSS software v28 (IBM). The Mann-Whitney *U* test, comparing fraction of immune cells and fibroblasts in organoid cultures with different invasive morphologies and comparing inverse circularity of organoids with and without stimulatory ligands (IL-6, CXCL12, MMP9, and TGF-β1), was implemented in R version 4.0.4 (https://cran.r-project.org/).

### Study approval.

This study was approved by the Institutional Review Boards of the Johns Hopkins University Hospital and the University of Mainz Medical Center. All patients provided written informed consent.

### Data availability.

RNA-seq data are available via direct request from the corresponding author.

## Author contributions

YJJ, HK, FS, and LDW designed the study. YJJ, FS, MFW, EH, TN, SD, BNS, PC, and MMG contributed to experimental work and acquisition of data. YJJ, HK, FS, MGL, BKK, JWZ, and GSO contributed to analysis of data. JRE, MTL, EJF, AJE, JSB, and LDW contributed to interpretation of data. YJJ, HK, FS, and LDW wrote the manuscript. All authors reviewed and approved the final manuscript. The order of co–first authors was determined by the temporal order of their involvement in the study.

## Supplementary Material

Supplemental data

Supplemental table 1

Supplemental table 2

Supplemental table 3

Supplemental table 4

Supplemental table 5

Supplemental table 6

Supplemental video 1

Supplemental video 2

Supplemental video 3

Supplemental video 4

Supplemental video 5

Supplemental video 6

## Version 1 03/07/2023

In-Press Preview

## Version 2 04/17/2023

Electronic publication
