# Supplementary material for: Morphology-guided transcriptomic analysis of human pancreatic cancer organoids reveals microenvironmental signals that enhance invasion
Source: J Clin Invest. 2023 Apr 17;133(8):e162054. doi: 10.1172/JCI162054 (PMC10104894; doi:10.1172/JCI162054)
Supplement: Supplemental data [file jci-133-162054-s031.pdf]

Supplemental figure 1

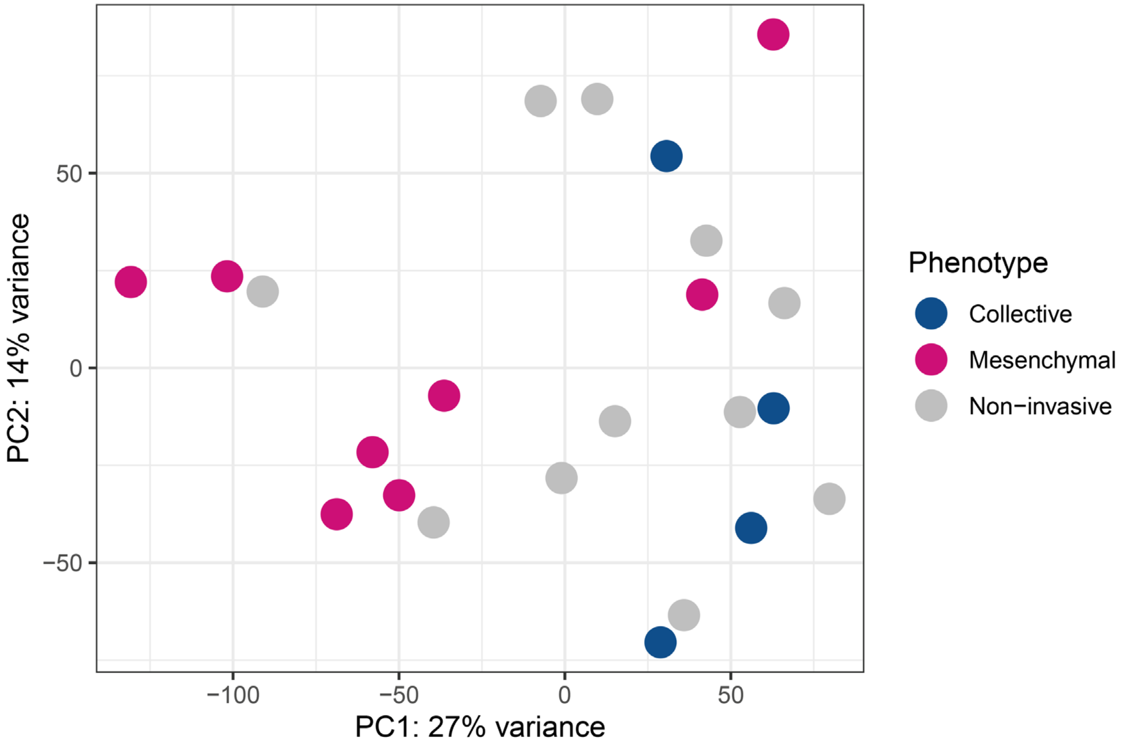

Supplemental figure 1. Principal component analysis of all invasive PDAC organoids and matched non-invasive organoids Principal component analysis shows clustering of the matched invasive and non-invasive organoids from the same primary tumors and also segregates by the invasive morphology as indicated.

Supplemental figure 2

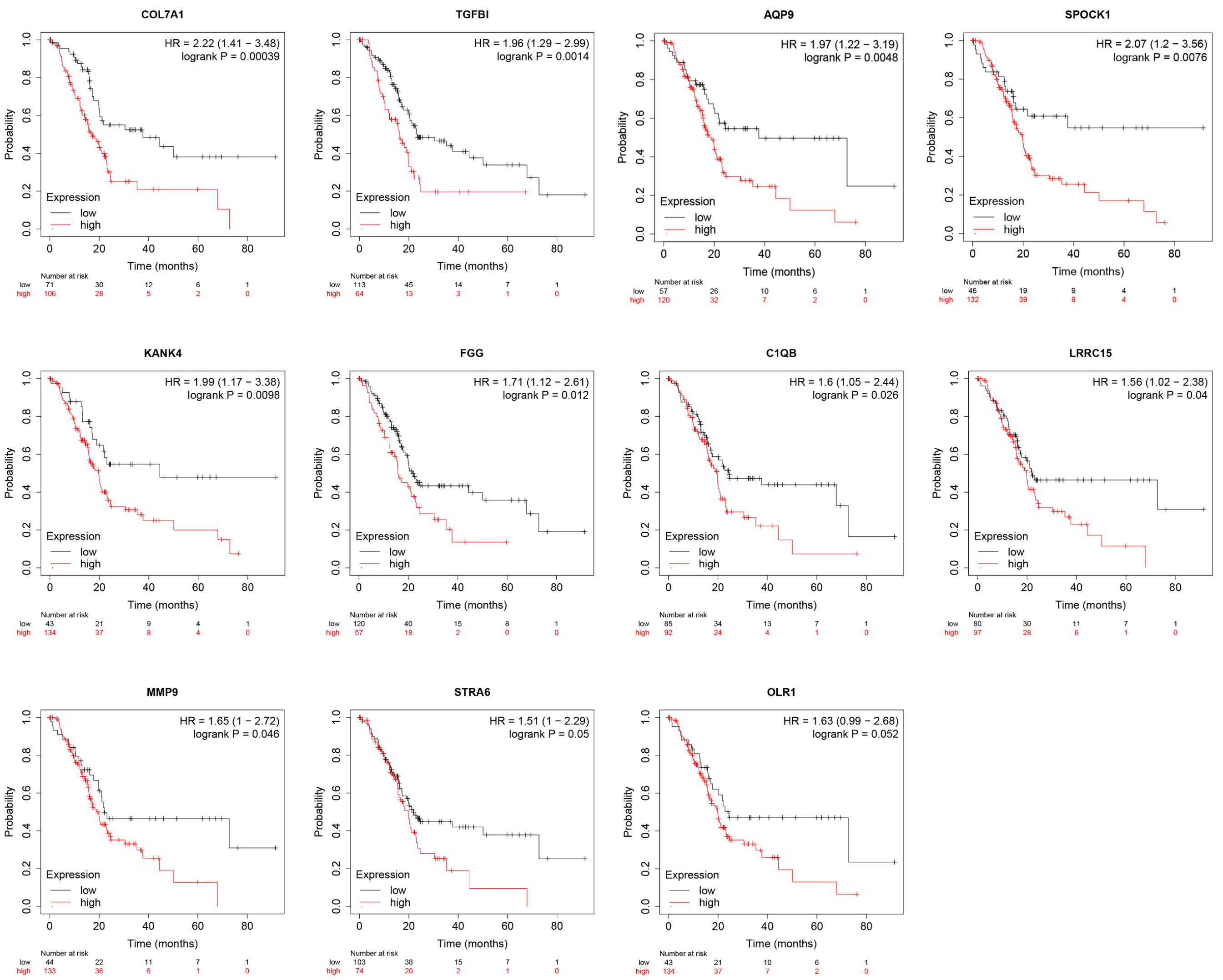

Supplemental figure 2. Kaplan Meier curves of TCGA PDAC cohort based on expression levels of 11 shared DE genes Kaplan Meier curves show statistically significant differences in survival time between tumors showing low and high quartile expression of indicated DE genes upregulated in invasive organoids ($p<0.05$).

Supplemental figure 3

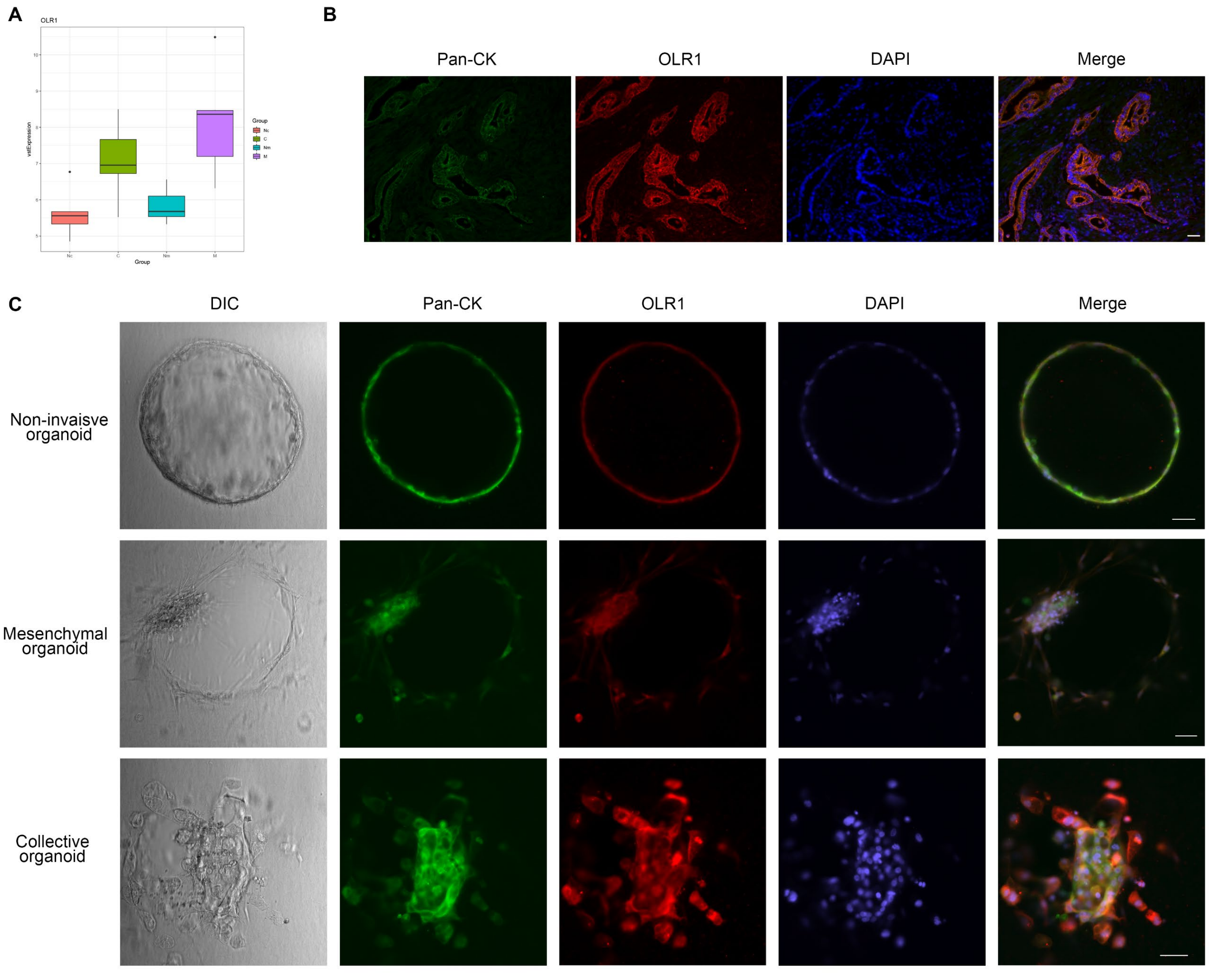

Supplemental figure 3 - OLR1 Immunofluorescence assay on PDAC organoids and tissue section (A) A bar plot of OLR1 mRNA expression in indicated organoid groups. (B) Immunofluorescence images of Pan-CK, OLR1 and DAPI staining in primary PDAC tissue sections. Scale bar: 50μm (C) Phase and immunofluorescence images of Pan-CK, OLR1 and DAPI staining in invasive and non-invasive organoids. Scale bars: 50μm

Supplemental figure 4

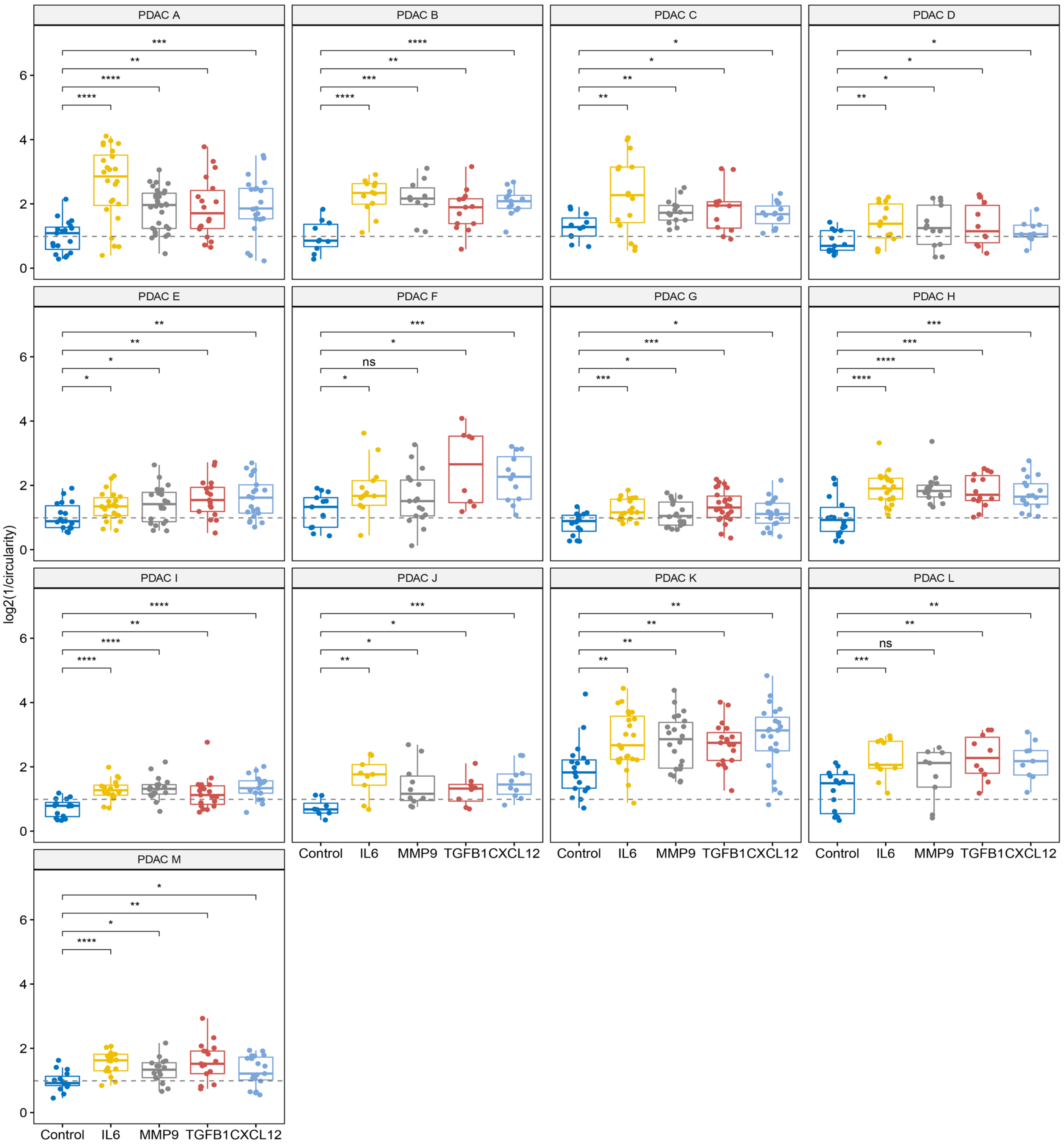

Supplemental figure 4 – Inverse circularity of PDAC organoid cultures after ligand treatment. Organoid invasion (log2(1/circularity)) analysis for control and ligand-treated cultures from twelve PDACs. Organoids derived from each PDAC are shown separately, with cultures labeled as A to M. One PDAC produced organoids with both invasive phenotypes; each phenotype from this culture was analyzed separately in graph K (collective) and graph L (mesenchymal) (ns>0.05, *<=0.05, **<=0.01, ***<=0.001, ****<=0.0001, Mann-Whitney U test).
